# SAT1/ALOX15 Signaling Pathway Is Involved in Ferroptosis After Skeletal Muscle Contusion

**DOI:** 10.3390/ijms252011317

**Published:** 2024-10-21

**Authors:** Huihuang Yang, Yingmin Li, Weihao Zhu, Xiaowei Feng, Hongjian Xin, Hao Chen, Guozhong Zhang, Min Zuo, Bin Cong, Weibo Shi

**Affiliations:** Collaborative Innovation Center of Forensic Medical Molecular Identification, Hebei Key Laboratory of Forensic Medicine, Department of Forensic Medicine, Hebei Medical University, Shijiazhuang 050017, China; 22033100274@stu.hebmu.edu.cn (H.Y.); 16000557@hebmu.edu.cn (Y.L.); 22031100049@stu.hebmu.edu.cn (W.Z.); 22033100268@stu.hebmu.edu.cn (X.F.); 22033100279@stu.hebmu.edu.cn (H.X.); 22033100283@stu.hebmu.edu.cn (H.C.); zhangguozhong@hebmu.edu.cn (G.Z.); 16700578@hebmu.edu.cn (M.Z.)

**Keywords:** skeletal muscle, contusion, ferroptosis, SAT1, ALOX15

## Abstract

Skeletal muscle contusion (SMC) is common in daily life and clinical practice, but the molecular mechanisms underlying SMC healing are unclear. Ferroptosis, a regulated cell death type, has gained attention recently. We observed iron overload in skeletal muscle following contusion through HE and Perls staining. Abnormal iron levels are highly likely to induce ferroptosis. Therefore, we aimed to explore whether iron overload after contusion leads to ferroptosis in skeletal muscle and the underlying mechanisms, which will help us understand the effects of iron abnormalities on skeletal muscle repair. Initially, we searched SMC gene expression profiles from the GEO database and used bioinformatics analysis to reveal ferroptosis occurrence. Then, we identified the gene *sat1* plays an important role in this process. We further established a rat SMC model and treated rats with ferroptosis inhibitors (Ferrostatin-1, Deferoxamine). Our findings confirmed iron overload from SMC can lead to ferroptosis in rats. We also demonstrated that SAT1 can regulate ferroptosis by affecting ALOX15. Moreover, we constructed a ferroptosis L6 cell model and found that SAT1 knockdown significantly inhibited ALOX15 expression and reduced cellular lipid peroxidation. In conclusion, these results indicated ferroptosis can occur following SMC, and SAT1, as a key regulator, affects skeletal muscle injury healing by mediating high ALOX15 expression, which in turn regulates lipid peroxidation.

## 1. Introduction

The human body contains skeletal muscle, a major and widespread subcutaneous tissue, making up about 40% of total body weight and storing 50–75% of body protein [1]. However, this tissue is susceptible to injury during exercise, with SMC being a common injury, particularly in violent cases [1,2]. SMC refers to muscle and connective tissue injuries caused by direct, blunt, and compressive force on skeletal muscle. It is characterized by secondary injuries such as muscle fiber breakage, inflammation, and fibrosis [3,4]. Skeletal muscle has a strong ability to repair itself, and the repair process after injury can be divided into three phases: the inflammatory damage phase, the anti-inflammatory repair phase, and the tissue remodeling phase [5,6]. These phases involve degeneration, inflammation, regeneration, and fibrosis, and do not occur independently but overlap and intertwine [7]. The mechanism of action for each phase is intricate and complex. Despite being clinically diagnostic and used in forensic identification, there are fewer studies on the molecular changes and mechanisms involved in the repair process of SMC.

Iron is a vital trace element in the human body, required for the function of numerous hemoproteins and non-heme iron proteins as a cofactor. It plays essential physiological roles, including erythropoiesis, oxygen transport, metabolism, DNA synthesis, and electron transfer [8]. Moreover, iron is crucial in oxidative stress. However, excessive iron accumulation can lead to toxic effects and multi-system diseases, impairing organ function [9,10]. Ferroptosis, a recently recognized form of iron-dependent regulated cell death, is characterized by lipid reactive oxygen species (ROS) accumulation due to abnormal iron ions [11]. Ferroptosis differs from other known cell death mechanisms, such as apoptosis, pyroptosis, necrosis, and autophagy [12]. Growing evidence suggests that ferroptosis participates in skeletal muscle aging and various diseases, significantly impacting patients’ physical function and quality of life [13,14]. For example, research showed that after exertional heat stroke, Rhabdomyolysis mice exhibit increased iron content, alterations in ferroptosis signature proteins, and characteristic mitochondrial morphology indicative of ferroptosis in skeletal muscle [15]. Huang Y et al. demonstrated that the accumulation of lipid peroxidation and iron overload induces myocyte ferroptosis through the p53/SLC7A11 pathway in the pathogenesis of sarcopenia [16]. However, the specific role of ferroptosis in the repair process of SMC has not been thoroughly investigated.

In the present study, we found that vessel ruptures in the tissue after SMC led to significant hemoglobin and transferrin accumulation, carrying iron ions and resulting in iron overload and subsequent induction of ferroptosis in the damaged tissue. Retrieving gene expression profiles of skeletal muscle contusion from the GEO database also showed ferroptosis after contusion. This study aims to investigate if iron overload after contusion leads to skeletal muscle ferroptosis and its regulatory mechanisms. Our goal is to elucidate the impact of abnormal iron levels on skeletal muscle repair from a new perspective.

## 2. Results

### 2.1. Iron Overload and DEGs Analysis After SMC

**Figure 1:**
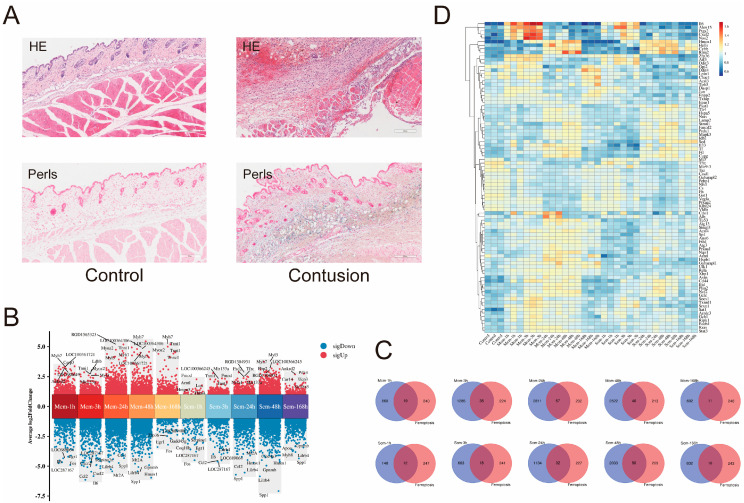
Tissue iron overload and analysis of ferroptosis-related genes following SMC. (**A**) Representative images of SMC at 24 h post-injury, stained with HE and Perls. Scale bars: 200 μm. (*n* = 3). (**B**) Volcano plot showing upregulated and downregulated DEGs. (**C**) Venn diagram representing the intersection of DEGs at each time point post-contusion with ferroptosis-related genes. (**D**) Heatmap of ferroptosis-related gene expression after contusion.

To determine iron overload, we constructed a rat model of SMC for 24 h. HE staining revealed significant hemorrhaging and hemosiderin in the subcutaneous tissue after SMC and Perl’s staining indicated high Fe^3+^ levels in the surrounding tissue after contusion (Figure 1A), suggesting that SMC may induce ferroptosis. To further investigate ferroptosis after SMC, we analyzed the rat skeletal muscle contusion transcriptomic dataset (GSE162565). Using adjusted *p*-value < 0.05 and |log2FC| > 1 as criteria, we identified differentially expressed genes (DEGs) at 1, 3, 24, 48, and 168 h post-mild and severe contusion. Volcano plot analysis showed upregulated and downregulated genes after contusion (Figure 1B). We intersected the DEGs with ferroptosis-related genes. The number of ferroptosis-related genes involved increased gradually within 24 h after mild contusion and within 48 h after severe contusion (Figure 1C). The heatmap showed ferroptosis-related gene expression changes during the healing process (Figure 1D).

### 2.2. SMC, Ferroptosis, and SAT1 Are Significantly Correlated

**Figure 2:**
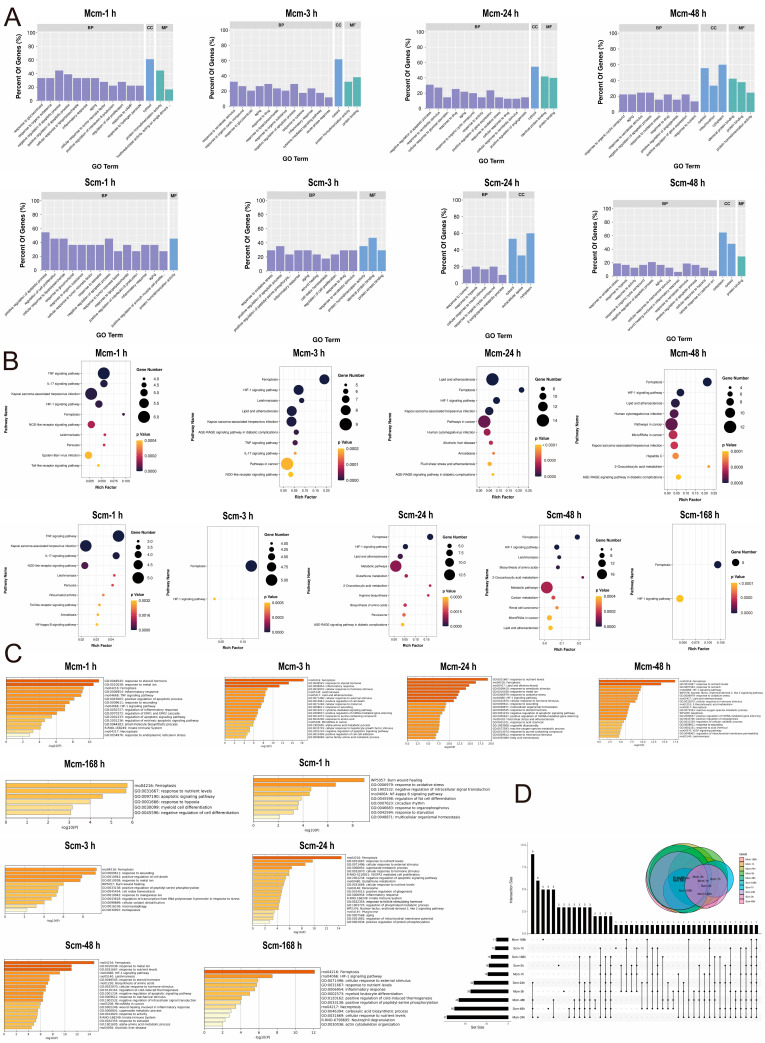
Enrichment analysis of SMC-ferroptosis DEGs and identification of Hub-DEGs. (**A**) GO functional enrichment analysis. Contents of ellipses in the figure: Mcm-1h: “oxidoreductase activity, acting on single donors with incorporation of molecular oxygen, incorporation of two atoms of oxygen”; Scm-1h: positive regulation of smooth muscle cell proliferation; Scm-3h: positive regulation of peptidyl-serine phosphorylation. (**B**) KEGG pathway enrichment analysis. (**C**) Metascape functional and pathway enrichment analysis. (**D**) The upset plot indicates that only SAT1 consistently shows differential expression in the ferroptosis process after contusion. The lines connecting the dots in the matrix on the lower right indicate the intersection of different sets.

Gene Ontology (GO) analysis of SMC-ferroptosis DEGs revealed both mild and severe contusions are in the inflammatory damage and repair stages, primarily enriching for functions such as inflammatory response, oxidative stress, and wound healing (Figure 2A). Kyoto Encyclopedia of Genes and Genomes (KEGG) pathway enrichment analysis showed the ferroptosis pathway involved in the skeletal muscle healing process (Figure 2B). To ensure result accuracy, we performed an additional round of enrichment analysis using Metascape, confirming ferroptosis pathway involvement in the SMC healing process (Figure 2C). To identify Hub-DEGs inducing ferroptosis after SMC, we constructed the temporal upset plot. The results showed that among ferroptosis-related genes, only *sat1* maintained consistently upregulated expression across all time groups following both mild and severe contusions (Figure 2D). Spermidine/spermine N1-acetyltransferase 1 (SAT1) is a rate-limiting enzyme controlling cellular polyamine catabolism and is highly expressed in ferroptosis [17,18]. In summary, these results suggest SMC induces ferroptosis, and SAT1, as a Hub-DEG, plays an indispensable role in regulating ferroptosis in skeletal muscle.

### 2.3. Ferroptosis Occurs After SMC of Rats 

**Figure 3:**
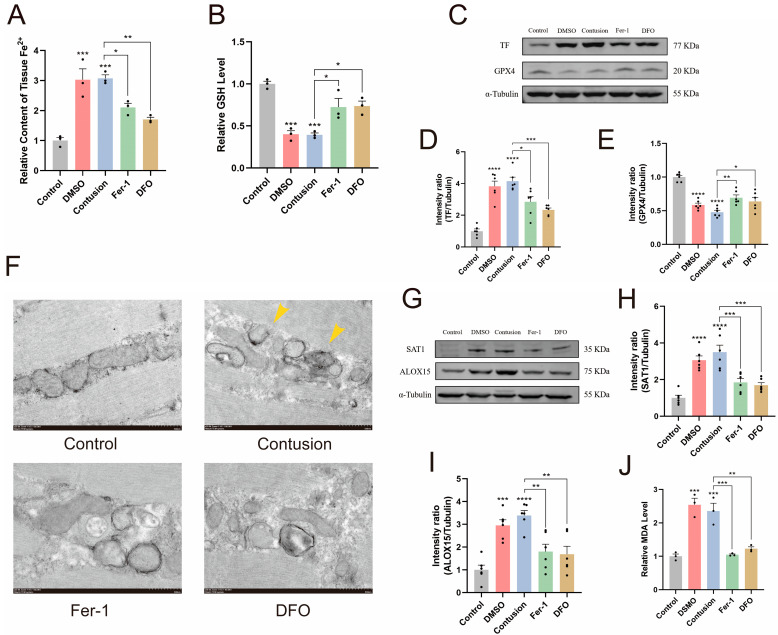
SMC leads to ferroptosis in rats. (**A**) Accumulation of Fe^2+^ after SMC. (*n* = 3). (**B**) Reduction in tissue reductase activity after SMC. (*n* = 3). (**C**–**E**) Western blotting for the expression of TF, GPX4, and α-Tubulin. (*n* = 6). (**F**) Representative TEM images of control, contusion, and ferroptosis inhibitor groups (Fer-1, DFO). Yellow arrows indicate abnormal mitochondria. Scale bar = 500 nm. (*n* = 3). (**G**–**I**) Western blotting shows the correlation of SAT1 and ALOX15 expression during ferroptosis after SMC. (*n* = 6). (**J**) Increased degree of lipid peroxidation after SMC. (*n* = 3). In the results above, treatment with ferroptosis inhibitors (Fer-1 and DFO) significantly improved the ferroptosis-related trends after contusion. Data are expressed as mean ± SEM. * *p* < 0.05, ** *p* < 0.01, *** *p* < 0.001, **** *p* < 0.0001.

To validate bioinformatics analysis results and determine the occurrence of ferroptosis after SMC, we separately constructed 24 h models of mild skeletal muscle contusion for control rats and rats injected with ferroptosis inhibitors (Fer-1 or DFO). Based on the speculation that tissue iron overload is the root cause of ferroptosis induced by SMC, and Fe^3+^ entering the cell will be reduced to Fe^2+^, we measured the Fe^2+^ levels. The results indicate that Fe^2+^ levels in the skeletal muscle tissue significantly increased after contusion; in contrast, treatment with Fer-1 and DFO reduced the formation of Fe^2+^ (Figure 3A). We also measured the levels of glutathione (GSH), which is important for the cellular antioxidant function during ferroptosis. As shown in Figure 3B, the GSH levels in the skeletal muscle significantly decreased after contusion. But, this reduction was significantly reversed after treatment with Ferrostatin-1 (Fer-1) and Deferoxamine (DFO). Transferrin (TF) and glutathione peroxidase 4 (GPX4) are considered important functional proteins in regulating the ferroptosis process. Western blotting results indicated that the levels of TF and GPX4 proteins in the skeletal muscle significantly decreased after contusion, but this change was reversed after treatment with Fer-1 and DFO (Figure 3C–E). Transmission electron microscopy (TEM) results revealed that compared to the control group, skeletal muscle cells following contusion exhibited mitochondrial shrinkage and increased membrane density, hallmarks of ferroptosis. However, treatment with Fer-1 and DFO significantly suppressed this effect (Figure 3F). In summary, these results indicated that ferroptosis occurs in SMC.

Although bioinformatics analysis has revealed that SAT1 plays a vital role in ferroptosis after SMC, the mechanism by which SAT1 regulates ferroptosis in skeletal muscle is still poorly understood. Studies have shown that in cancer cells, SAT1 expression correlates with arachidonate 15-lipoxygenase (ALOX15) [19]. ALOX15 is involved in oxidative stress-related cell death and is crucial for forming lipid peroxides [20]. Interestingly, heatmap analysis revealed that ALOX15 expression also increased after SMC (Figure 1D). However, the regulatory mechanism of SAT1 and ALOX15 in the process of ferroptosis induced by SMC is not yet clear. Therefore, this study hypothesizes that in ferroptosis induced by SMC, SAT1 mediates the high expression of ALOX15 to regulate the lipid peroxidation process in skeletal muscle. We examined SAT1 and ALOX15 protein expression using Western blotting. Results showed SAT1 expression increased in ferroptosis after SMC, with ALOX15 expression trends consistent with SAT1 (Figure 3G–I). Since ALOX15 overexpression leads to lipid peroxidation, we measured malondialdehyde (MDA) levels, a marker of lipid peroxidation. Results indicated lipid peroxidation product accumulation (Figure 3J). In the above experiments, changes observed after SMC were significantly improved by treatment with Fer-1 or DFO.

### 2.4. RSL3 Induces Ferroptosis in L6 Cells 

**Figure 4:**
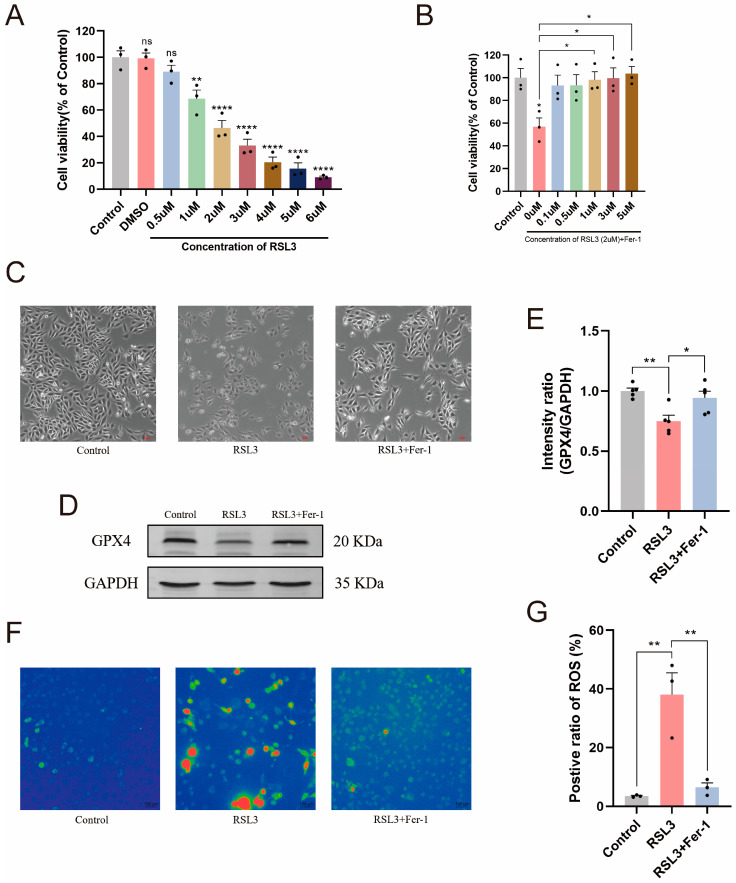
RSL3 induces damage in L6 cells, which can be reversed by Fer-1. (**A**–**C**) Exposure of L6 cells to various concentrations of RSL3 for 24 h resulted in a dose-dependent decrease in cell viability, which was significantly rescued by Fer-1 treatment. Scale bar = 100 μm. (*n* = 3). (**D**,**E**) Western blotting showed that the expression of GPX4 decreased in L6 cells induced by RSL3. (*n* = 5). (**F**,**G**) Representative fluorescence images of ROS measured by DCFH-DA. Scale bar = 100 μm. (*n* = 3). Data are expressed as mean ± SEM. ns: no significant, * *p* < 0.05, ** *p* < 0.01, **** *p* < 0.0001.

To validate our hypothesis, we constructed a ferroptosis model using the L6 rat myoblast cell line in vitro. We assessed whether RSL3 exposure could induce ferroptosis in L6 cells by measuring cell viability using the CCK-8 assay. As shown in Figure 4A, exposure of L6 cells to 0.5, 1, 2, 3, 4, 5, and 6 μmol/L of RSL3 for 24 h resulted in a dose-dependent decrease in cell viability, confirming the toxic effects of RSL3 on L6 cells. Based on this result, we selected 2 μmol/L of RSL3 as the appropriate concentration for subsequent experiments, as the cell viability was significantly reduced to approximately 50% compared to the control group. Next, to determine whether Fer-1 could rescue L6 cell damage induced by RSL3, we added 0.1, 0.5, 1, 3, and 5 μmol/L of Fer-1 along with 2 μmol/L RSL3 to the L6 cells. The cell viability results showed that, compared to those treated with RSL3 alone, Fer-1 attenuated the RSL3-induced damage in a dose-dependent manner. We selected 3 μmol/L of Fer-1 as the appropriate concentration for subsequent experiments (Figure 4B,C). Furthermore, to confirm that RSL3-induced L6 cell damage was caused by ferroptosis, we detected the level of GPX4, an important functional protein in ferroptosis. Western blot results indicated that RSL3 induced low expression of GPX4 in L6 cells (Figure 4D,E). ROS accumulation is closely associated with ferroptosis [11,21]. Therefore, our results from observing DCFH-DA uptake showed that, compared to untreated normal cells (Figure 4F,G), the changes were significantly improved by Fer-1. In summary, these results indicate that RSL3 induced ferroptosis in L6 cells.

### 2.5. The SAT1/ALOX15 Signaling Pathway Regulates Ferroptosis in Skeletal Muscle

**Figure 5:**
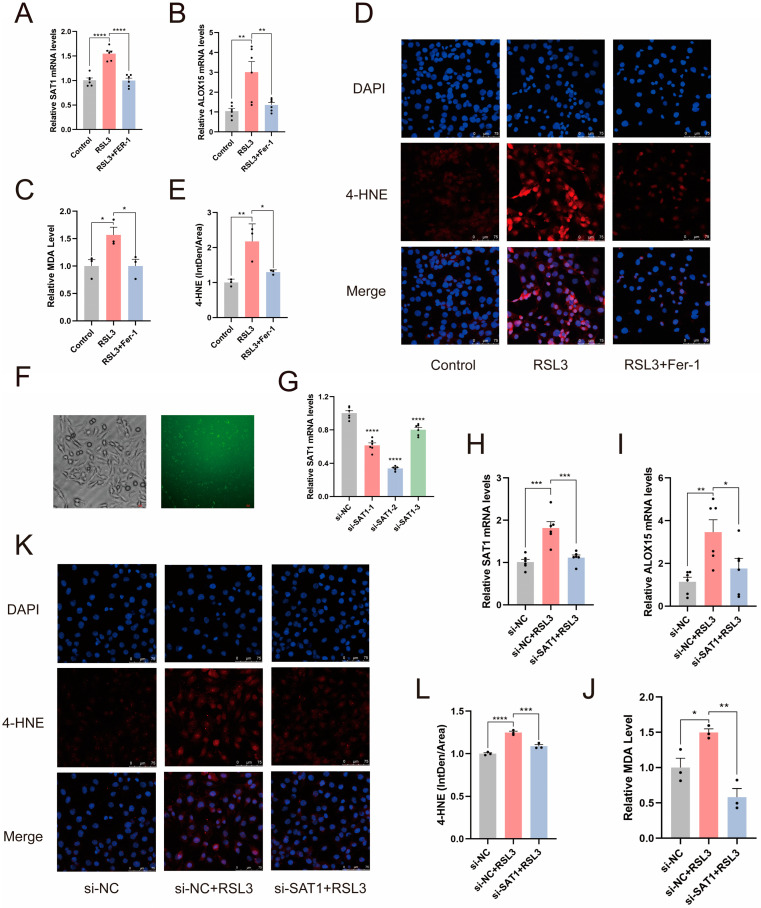
The SAT1/ALOX15 signaling pathway regulates the molecular mechanism of RSL3-induced ferroptosis in L6 cells. (**A**,**B**) RT-qPCR showed that RSL3 induced high expression of SAT1 and ALOX15 in L6 cells. (*n* = 6). (**C**–**E**) MDA level detection and 4-HNE staining indicated an increase in the degree of lipid peroxidation in L6 cells induced by RSL3. Scale bar = 75 μm. Red fluorescence: 4-HNE; blue fluorescence: DAPI. (*n* = 3). The above changes induced by RSL3 were significantly inhibited by Fer-1 treatment. (**F**) The transfection efficiency of FITC was observed. Left: bright field image; right: FITC image. Scale bar = 100 μm. (**G**) RT-qPCR was used to evaluate the knockdown efficiency of si-SAT1. (*n* = 6). (**H**,**I**) RT-qPCR indicated that SAT1 knockdown inhibits ALOX15 expression in ferroptosis. (*n* = 6). (**J**–**L**) MDA level detection and 4-HNE staining showed that SAT1 knockdown alleviates the degree of lipid peroxidation in ferroptosis. Scale bar = 75 μm. (*n* = 3). Data are expressed as mean ± SEM. * *p* < 0.05, ** *p* < 0.01, *** *p* < 0.001, **** *p* < 0.0001.

Subsequently, we demonstrated that RSL3 significantly increased SAT1 and ALOX15 mRNA levels in L6 cells (Figure 5A,B). We assessed lipid peroxidation in L6 cells by detecting MDA and 4-hydroxynonenal (4-HNE), lipid peroxidation products. As shown in Figure 5C–E, RSL3 induced accumulation of lipid peroxidation products in L6 cells. These changes were significantly reduced by Fer-1 treatment. These results indicated high SAT1 and ALOX15 expression during ferroptosis in L6 cells. To determine whether SAT1 mediates ALOX15 expression to regulate lipid peroxidation in skeletal muscle ferroptosis, we evaluated SAT1 knockdown on ferroptosis in L6 cells. We assessed the transfection efficiency of SAT1 by transfecting fluorescent probes, and RT-qPCR indicated Si-SAT1-2 had the highest knockdown efficiency (≈65%), so we selected it for subsequent experiments (Figure 5F,G). As shown in Figure 5H, SAT1 mRNA level significantly increased in RSL3-induced L6 cells, while SAT1 was significantly reduced after SAT1 knockdown. Similarly, the ALOX15 mRNA level was consistent with SAT1, and the ALOX15 mRNA level in RSL3-induced L6 cells significantly decreased after SAT1 knockdown compared to RSL3 treatment alone (Figure 5I). These results suggest SAT1 acts as an upstream factor mediating ALOX15 expression in L6 cells ferroptosis. Since ALOX15 is closely related to lipid peroxidation, to assess the impact of SAT1/ALOX15 signaling on lipid peroxidation in RSL3-induced L6 cells, we again measured lipid peroxidation products in L6 cells after different treatments. As shown in Figure 5J–L, RSL3 induced an increase in MDA and 4-HNE levels in L6 cells, whereas SAT1 knockdown significantly suppressed lipid peroxidation. In conclusion, SAT1 mediates high ALOX15 expression and regulates the lipid peroxidation process in L6 cell ferroptosis.

## 3. Discussion

With increasing research on the role of ferroptosis in skeletal muscle pathological conditions, the importance of ferroptosis in SMC has risen [22]. In this study, we initially inferred the occurrence of ferroptosis following SMC through bioinformatics analysis and identified SAT1 as a hub-DEG continuously functioning in the ferroptosis of the healing phase after contusion. Subsequently, we established a rat model of SMC and validated our hypothesis in vivo, discovering a significant correlation between the expression of SAT1 and ALOX15 in skeletal muscle ferroptosis. Moreover, we constructed a ferroptosis model in L6 cells induced by RSL3 in vitro, and SAT1 knockdown suppressed ALOX15 expression and mitigated lipid peroxidation levels. Therefore, we conclude that SMC induces ferroptosis, and the SAT1/ALOX15 signaling pathway regulates the lipid peroxidation process of ferroptosis in skeletal muscle.

Ferroptosis is primarily iron-dependent and characterized by lipid peroxidation [23]. Intriguingly, we found blood vessels in the subcutaneous tissue ruptured after SMC, leading to significant iron (heme-containing red blood cells and Fe^3+^-carrying TF) leakage into the contused tissue and subsequently into the skeletal muscle cells. The intracellular Fe^3+^ is reduced to Fe^2+^ by the prostate six-transmembrane epithelial antigen 3 (STEAP3), and then incorporated into the labile iron pool (LIP) and stored as ferritin [24,25]. Notably, the unstable, highly reactive Fe^2+^ in the LIP generates the production of hydroxyl radicals (•OH) through the Fenton reaction, which can directly react with polyunsaturated fatty acids (PUFAs) in the cell membrane, producing abundant lipid ROS and triggering ferroptosis [26,27]. Lipid ROS includes lipid alkoxyl radicals (L-O•), lipid peroxyl radicals (L-OO•), and lipid peroxides (L-OOH), mainly produced through non-enzymatic autoxidation or enzymatic lipid peroxidation pathways, with the iron-dependent Fenton reaction playing a crucial role [9,28]. Our evidence indicates that the MDA levels (one of the main products of lipid peroxidation) are increased in skeletal muscle under iron overload. Additionally, GSH and GPX4 are vital regulatory factors in the cellular antioxidant defense pathway [29]. Our results suggest that there is a depletion of GSH and a decrease in the activity of GPX4 after SMC, which further leads to the accumulation of lipid peroxides because they are not effectively metabolized by the GPX4-catalyzed reduction reaction.

Polyamines are polycationic aliphatic amines that bind strongly to nucleic acids and participate in various biological processes, including DNA replication, transcription, translation, and the regulation of intercellular communication [30,31]. SAT1 is an important regulatory factor in the catabolic metabolism of polyamines, and the polyamine metabolite N1-acetylspermidine can serve as a substrate for histone acetylation to guide chromatin modification [32,33]. An increasing number of studies have shown that SAT1 is involved in the ferroptosis process of different diseases such as Alzheimer’s disease, lung adenocarcinoma, and neuropathic pain [34,35,36]. The enzymatic lipid peroxidation pathway involves the lipoxygenase (LOX) enzyme using PUFAs as substrates to participate in the formation of lipid peroxides (L-OOH), which can cause damage to cell and organelle membranes [37,38]. Notably, in 2016, Ou Y et al. discovered that P53-mediated SAT1 was associated with the expression level of ALOX15 in cancer cells [19]. Our study found that in rat SMC-induced ferroptosis, the protein levels of SAT1 and ALOX15 were highly expressed. We further confirmed that knocking down SAT1 led to a decrease in ALOX15 mRNA levels and attenuated the degree of lipid peroxidation in vitro experiments. Based on these results, we conclude that ferroptosis occurs after SMC, in which SAT1 plays a key role and regulates the lipid peroxidation process by mediating the high expression of ALOX15 in skeletal muscle.

As research into ferroptosis progresses, inhibiting ferroptosis is considered a promising approach for treating many diseases. For instance, Fer-1 alleviated Ang II-induced ferroptosis in vascular smooth muscle cells and delayed the formation of aortic aneurysms [39]; DFO treatment improved muscle regeneration capacity in pressure injuries of muscle [40]. In our study, the ferroptosis inhibitors Fer-1 and DFO effectively suppressed the progression of ferroptosis after SMC in rats, including mitigating iron overload, enhancing antioxidant capacity, and reducing the accumulation of lipid peroxidation products. Notably, research has implicated the ACSL4-SAT1 pair in the regulation of ferroptosis and inflammatory responses in rotator cuff tears [41]. Similarly, we observed increased SAT1 expression during ferroptosis induced by SMC and demonstrated that knocking down SAT1 significantly protected L6 myoblasts from RSL3-induced ferroptosis in vitro. This suggests that targeting SAT1 to inhibit ferroptosis may be a novel therapeutic strategy for preventing secondary injury after contusion. Additionally, our bioinformatics results indicated that the inflammatory response after SMC may be associated with ferroptosis, prompting further investigation of the impact of inflammation on ferroptosis.

However, it is undeniable that this study has some limitations. Firstly, we have demonstrated the association between SMC and ferroptosis, but we did not explore whether other modes of cell death are involved, including autophagy, apoptosis, and pyroptosis. secondly, we have not yet clarified the regulatory mechanism by which SAT1 mediates the expression of ALOX15 in skeletal muscle. Consequently, these limitations need to be addressed in subsequent experiments.

## 4. Materials and Methods

### 4.1. Data Acquisition and Processing

Gene expression profiles of SMC were obtained from the NCBI-GEO database (GSE162565). There were three control rat samples, 15 mildly contused rat samples, and 15 severely contused rat samples (the latter divided into five subgroups: 1, 3, 24, 48, and 168 h post-contusion, each with three samples, totaling 33 samples. DEGs were identified using the GEO2R analysis tool (GEO2R - GEO - NCBI, https://www.ncbi.nlm.nih.gov/geo/geo2r/?acc=GSE162565; accessed on 12 November 2023), with DEG criteria set at |log2FC| > 1 and adjusted *p*-value < 0.05.

### 4.2. Enrichment Analysis of Ferroptosis-Associated Genes and Identification of Hub-DEGs

SMC-ferroptosis DEGs were identified by intersecting previously obtained SMC DEGs with ferroptosis-associated genes from the FerrDb website (http://www.zhounan.org/ferrdb/legacy/index.html; accessed on 14 November 2023). The DAVID database (DAVID Functional Annotation Bioinformatics Microarray Analysis, https://david.ncifcrf.gov/; accessed on 15 November 2023) was utilized for GO and KEGG enrichment analysis of SMC-ferroptosis DEGs. Correlation analysis was performed as previously described [42]. Pathway enrichment analysis was also performed using Metascape (http://metascape.org/gp/index.html; accessed on 16 November 2023). Upsets were generated using the OmicStudio tools (https://www.omicstudio.cn/tool; accessed on 20 November 2023) and ferroptosis-associated genes in each time group after contusion were intersected again to screen for Hub-DEGs involved in ferroptosis after SMC. A significance level of *p* < 0.05 was applied.

### 4.3. Chemical Reagents and Pharmaceuticals

Dimethyl Sulfoxide (DMSO; TCI America, Portland, OR, USA, D0798), Ferrostatin-1 (Cayman Chemical, Ann Arbor, MI, USA, 17729), and Deferoxamine (Cayman Chemical, 14595) were dissolved in DMSO at a concentration of 5 mg/mL. (1*S*,3*R*)-RSL3 (Cayman Chemical, 19288) was dissolved in DMSO at an approximate concentration of 20 mg/mL. All drugs were diluted with PBS to ensure DMSO concentration was less than 10% before use.

### 4.4. Animal Model of SMC

Sprague Dawley healthy male rats, weighing 220–250 g, were purchased from Beijing Vital River Laboratory Animal Technology Co., Ltd. (Beijing, China). Rats were housed in cages with food and water in a temperature-controlled room (22–24 °C) with a relative humidity of 40–60% for a 12 h light-dark cycle. Forty rats were randomly divided into Control, DMSO, Contusion, Fer-1, and DFO groups. SMC model: a 500 g spherical weight (diameter, 0.8 cm) was free-fallen from a height of 15 cm through a transparent plexiglass tube onto the thigh muscle of the hindlimb, transferring ~0.74 J of energy (calculated as E = mgh) to the limb [2]. Rats were euthanized 24 h post-contusion, and skeletal muscle tissue from the thigh was collected. Control group (n = 8): no treatment was performed before tissue collection; DMSO group (n = 8): 5% DMSO solvent (5 mL/rat) was injected intraperitoneally into rats before contusion; Contusion group (n = 8): no treatment was performed before contusion; Fer-1 group (n = 8): before contusion, rats were injected intraperitoneally with Fer-1 solvent (1.5 mg/rat); DFO group (n = 8): before contusion, rats were injected intraperitoneally with DFO solvent (50 mg/rat).

### 4.5. HE and Perls Staining

Skeletal muscle tissues were fixed in 4% paraformaldehyde for over 24 h. After dehydration with ethanol, tissues underwent wax dipping and paraffin embedding. Sections (4 μm thick) were cut and stained with HE and Perls stains, following standard procedures. Three sections of each rat (three rats per group) were observed and photographed under the microscope, with randomly selected fields of view around the contusion area imaged at ×100 magnification.

### 4.6. Detection of Fe^2+^, GSH, and MDA

We utilized the Tissue Iron Content Assay Kit (Boxbio, Beijing, China, AKIC001M), Total Glutathione Assay Kit (Beyotime, Beijing, China, S0052), and Lipid Peroxidation MDA Assay Kit (Beyotime, S0131S) to assay skeletal muscle tissues and cells according to the respective manufacturer’s instructions. We measured absorbance values at specific wavelengths using an enzyme marker.

### 4.7. TEM

Fresh skeletal muscle tissues were fixed with 2.5% glutaraldehyde at 4 °C overnight. TEM was utilized to observe and document morphological changes in rat skeletal muscle cells at a scale of 500 nm after different treatments.

### 4.8. Protein Extraction and Immunoblotting

The samples were added to RIPA lysis buffer (Solarbio, Beijing, China, R0010) containing protease inhibitors and then thoroughly ground in a low-temperature freeze grinder. After centrifuging at 4 °C for 10 min to obtain the supernatant, the total protein concentration was quantified using the BCA method. Equal amounts of protein lysates were separated by SDS-PAGE gel electrophoresis and transferred to PVDF membranes. The membranes were blocked with 5% BSA at room temperature for 1 h, then incubated overnight at 4 °C with different primary antibodies. After three washes with TBST, the membranes were incubated with secondary antibodies at room temperature for 1 h. The bands were visualized using a LI-COR Odyssey scanner (LI-COR, Lincoln, NE, USA). The relative expression of the target protein was analyzed using ImageJ 1.54g (National Institutes of Health, Bethesda, MD, USA). The primary antibodies used were: anti-GPX4 antibody (Huabio, Woburn, MA, USA, ET1706-45) (1:2000), anti-TF antibody (Proteintech, Rosemont, IL, USA, 17435-1-AP) (1:1000), anti-SAT1 antibody (Proteintech, 10708-1-AP) (1:2000), anti-ALOX15 antibody (Abcam, Cambridge, UK, AB244205) (1:1000), anti-α-Tubulin antibody (GeneTex, Irvine, CA, USA, GT114) (1:5000), anti-GAPDH antibody (Report Biotech, Somerset, NJ, USA, RA1005) (1:20,000).

### 4.9. Cell Culture

L6 rat myoblast cells (ZQ0140) were obtained from Zhong Qiao Xin Zhou Biotechnology (Shanghai, China). L6 cells were cultured in high glucose DMEM (Viva Cell BIOSCIENCES, Denzlingen, Germany, C3113-0500) supplemented with 10% fetal bovine serum (ExCell Bio, Shanghai, China, FSP500) and 1% Penicillin-Streptomycin Liquid (Solarbio, P1400) at 37 °C in a humidified incubator with 5% CO_2_.

### 4.10. Cell Viability Assay

Cell Counting Kit-8 (CCK-8) (Report Biotech, RP-RC3028) was utilized to assess cell viability after exposure to various treatments. L6 cells were seeded at 5000 cells/well in a 96-well plate. After the cells adhered and were treated with different drugs, 10 μL of CCK-8 solution was added to each well. The plate was then incubated at 37 °C for 1 h. The absorbance at 450 nm was measured using an enzyme marker.

### 4.11. ROS Assessment

L6 cells were seeded in 6 well plates at a density of 1.8 × 10^5^ cells/well and exposed to different experimental conditions. The Reactive Oxygen Species Assay kit (Report biotech, PK1002) was used according to the manufacturer’s instructions. After treatment with different conditions, cells were stained with 50 μM DCFH-DA. Inverted fluorescence microscopy was used to observe DCFH-DA uptake and assess intracellular ROS levels. The number of ROS-positive cells in each group was normalized to the control group by counting and measuring fluorescence intensity using ImageJ.

### 4.12. Real-Time Fluorescent Quantitative PCR

Total RNA was extracted from L6 cells using the chloroform extraction method and quantified by nucleic acid quantification. Reverse transcription was performed on 500 ng RNA into cDNA using the PromeScript^TM^ RT reagent Kit with gDNA Eraser (Perfect Real Time) (Takara, Kusatsu, Japan, RR047A). Quantitative PCR was then performed using TB GreenR^®^ Premix Ex Taq^TM^ II (Takara, RR820A) and a 7500 real-time fluorescent quantitative PCR system. Gene expression was normalized to GAPDH using the 2^−ΔΔCt^ method. GAPDH was obtained from Sangon Biotech, Shanghai, China, and the following primers were designed: SAT1 forward 5′-gtgatgagtgattaccgaggctttg-3′, SAT1 reverse 5′-gcagcgacacttcatagcaacc-3′; ALOX15 forward 5′-cttcctgcccgcctggtattc-3′, ALOX15 reverse 5′-ccgcttcaaacagagtgcctttc-3′.

### 4.13. Immunofluorescence

L6 cells were seeded in 24 well plates at 2 × 10^4^ cells/well and cultured in cell crawl media. After treatment with different conditions, cells were fixed with 4% paraformaldehyde for 30 min, then washed three times with PBS. PBST (PBS containing 1% Triton X-100) permeabilized cells for 20 min. After 40 min of blocking with goat serum, cells were incubated overnight with anti-4-HNE antibody (Bioss Antibodies, Woburn, MA, USA, bs-6313R) (1:200). Following three washes with PBS, cells were incubated for 1 h at 37 °C with secondary fluorescent antibodies. DAPI (Southern Biotech, Birmingham, AL, USA, 0100-20) was used to stain nuclei. Finally, cells were observed using a Leica SP8 confocal fluorescence microscope (Leica Microsystem, Wetzlar, Germany), and fluorescent intensity was measured using ImageJ. The mean fluorescent intensity for each group was normalized to the control group.

### 4.14. Cell Transfection

si-RNAs targeting SAT1, a negative control si-RNA, and a fluorescently labeled si-RNA were obtained from RiboBio, Guangzhou, China. Following the manufacturer’s instructions, we transfected L6 cells, which had reached approximately 30–50% confluence, using riboFECT^TM^ CP transfection reagent (RiboBio, R10035.7) for 48 h. The efficiency of si-RNA transfection and knockdown level were assessed by inverted fluorescence microscopy and RT-qPCR.

### 4.15. Statistical Analysis

All data analysis was conducted using GraphPad Prism software (version 8). The bars in the graphs indicate the standard error of the mean (SEM). Statistical significance was estimated by Normality and Lognormality Tests and One-way ANOVA. *p* < 0.05 was considered statistically significant. All experiments were repeated independently at least three times.

## Data Availability

The datasets generated during and/or analyzed during the current study are available from the corresponding author upon reasonable request.
